# Transforming Assessment of Behavioral Impairment: The Role of Facial AI Technologies

**DOI:** 10.1002/alz70856_102787

**Published:** 2025-12-26

**Authors:** Yuting Song, Bingjie Wu, Menghan Zhou, Xingyao Wang, TaiHou Tng, Adeline Su Lyn Ng, Shahul Hameed, Simon Kang Seng Ting, Tanvi Verma, Mingrui Tan, Jia Huang, Muhammad Hazwan Bin Ismail, Liangli Zhen, Xuling Lin, Fei Gao, Yee Leng Tan, Yong Liu, Eng King Tan, Siow Mong Rick Goh, Wing Lok Au, Kok Pin Ng

**Affiliations:** ^1^ Institute of High Performance Computing (IHPC), Agency for Science, Technology and Research (A*STAR), Singapore, Singapore; ^2^ National Neuroscience Institute, Singapore, Singapore; ^3^ Lead Innovation Catalyst, Lions Befrienders Service Association, Singapore, Singapore

## Abstract

**Background:**

Early detection of behavioral impairment is vital for timely intervention and improved patient outcomes. However, traditional assessment tools are limited by practice effects and lack of trained staff in busy clinics. Impaired behavioral symptoms are increasingly shown to be associated with physiological changes, inappropriate facial expressions and micro‐expressions. Hence, this study aims to develop a novel artificial intelligence (AI)‐based facial and physiological response analysis to facilitate the early detection of behavioral impairment among at‐risk individuals in the community.

**Method:**

We recruited 66 seniors aged 60 and above from a community cognitive screening program. We collected facial video samples at rest and during behavioral assessments using the Geriatric Depression Scale‐15 (GDS‐15). In this pilot study, we built AI algorithms to derive heart rate (HR) from the facial video samples and correlated the HR with the GDS‐15 scores.

**Result:**

Our in‐house AI‐based HR detection at rest and during behavioral assessment achieved a mean average error 10.1 and 13.9 beats per minute respectively. The HR of the participants are mean 71.4, SD 8.9, range 53‐95 and mean 69.5, SD 7.8, range 52‐85 at rest and during behavioral assessment respectively. The GDS‐15 of the participants are mean 1.2, SD 2.0, range 0‐9. The overall correlation of resting HR and HR during behavioral assessment with the GDS‐15 score is 0.03 and 0.15 respectively. We then stratified the cohort using the GDS‐15 scores into normal (0‐4) and depression (≥5). In the depression group, the correlations of HR with GDS‐15 are 0.87 (resting) and 0.98 (during assessment), which indicates that higher depressive symptoms are associated with higher HR both during resting and assessment. On the other hand, the correlations of HR with GDS‐15 in the normal group are 0.13 (resting) and 0.06 (during assessment), suggesting almost no relationship.

**Conclusion:**

Our preliminary findings support HR derived from facial AI technologies as a promising approach in assisting clinicians with screening of behavioral impairments. In the next steps, we will combine facial features and physiological measures derived from facial AI technologies to enhance the accuracy of our AI model in diagnosing behavioral impairment among at‐risk individuals.

